# Development and feasibility of the Community Ageing Research Across Ethnicities (CARE) Network APP for diverse communities and faith groups

**DOI:** 10.1002/alz70858_104972

**Published:** 2025-12-26

**Authors:** Kamara Israel‐McLeish

**Affiliations:** ^1^ King's College London, London, Great britan, United Kingdom

## Abstract

**Background:**

Dementia disproportionately affects ethnic minority communities, yet these populations remain underrepresented in research and clinical trials. The Community Ageing Research Across Ethnicities (CARE) app aims to address this disparity by enhancing awareness of dementia, facilitating research participation, and improving access to relevant services within diverse ethnic and faith communities. This study will evaluate the feasibility of the CARE app, assessing its effectiveness in increasing dementia knowledge, fostering trust in research, and improving awareness of mental health services. In alignment with the UK's ‘Dementia 2020 Plan', this study seeks to provide a scalable, culturally sensitive intervention to mitigate existing health disparities. By integrating evidence‐based resources with interactive engagement tools, the app has the potential to enhance dementia literacy, promote early intervention, and support greater inclusivity in dementia research. The findings from this study will inform future refinements and broader implementation of digital interventions for dementia awareness and research engagement.

**Method:**

This feasibility study will evaluate a 4‐week online intervention delivered via the app to older adults living in the UK. The study will assess the app's usability, acceptability, and effectiveness in improving dementia awareness and research engagement. The CARE app will be developed through public and patient involvement consultations to ensure cultural and linguistic appropriateness for diverse communities.

Pre and post‐intervention assessments will be conducted to measure changes in dementia awareness, research participation knowledge, and perceived barriers and facilitators to service use. The usability and acceptability of the app will be evaluated using standardised Likert scales and focus groups. Findings from this study will provide critical insights into the potential effectiveness of digital interventions in improving dementia awareness and promoting inclusivity in dementia research.

**Results:**

Study participant demographics will be reported, along with a feasibility analysis assessing the intervention's impact. Secondary measures, including dementia knowledge and research participation, will also be evaluated to determine broader benefits.

**Conclusion:**

Increasing dementia knowledge within ethnic populations has been shown to improve early detection and shift attitudes toward memory‐related discussions. The app hopes to help bridge the gap between communities and access to dementia information, research, and healthcare services through digital interventions, promoting inclusivity and early engagement.

